# The Effects of Okra Consumption on Glycemic Parameters and Lipid Profile in Adults: A Systematic Review and Meta‐Analysis

**DOI:** 10.1002/fsn3.4599

**Published:** 2024-11-20

**Authors:** Xiaolei Zhang, Jinxin Miao, Yagang Song, Mingsan Miao

**Affiliations:** ^1^ Academy of Chinese Medical Sciences Henan University of Chinese Medicine Zhengzhou China; ^2^ Henan Collaborative Innovation Center of Research and Development on the Whole Industry Chain of Yu‐Yao Henan University of Chinese Medicine Zhengzhou China

**Keywords:** glycemic parameters, lipid profile, meta‐analysis, okra, systematic review

## Abstract

The clinical research findings on the effects of okra consumption on blood glucose and lipids are inconsistent. In this study, we aimed to explore the impact of okra consumption on glycemic parameters and lipid profile in adults, including homeostatic model assessment for insulin resistance (HOMA‐IR), glycosylated hemoglobin A1c (HbA1c), fasting blood glucose (FBG), triglyceride (TG), total cholesterol (TC), low‐density lipoprotein cholesterol (LDL‐C), and high‐density lipoprotein cholesterol (HDL‐C). To find relevant randomized controlled trials (RCTs), we systemically searched EMBASE, Web of Science, PubMed, Cochrane Library, and Scopus until April 2024. According to the inclusion and exclusion criteria, eight studies involving 521 participants were ultimately included in the present study. Compared to placebo, okra consumption remarkably decreased FBG (WMD: −32.56 mg/dL; 95% CI: −48.83, −16.28; *p* < 0.001; *I* ^2^ = 84.7%), HbA1c (WMD: −0.48%; 95% CI: −0.81, −0.16; *p* = 0.004; *I* ^2^ = 5.5%), TG (WMD: −13.16 mg/dL; 95% CI: −23.54, −2.77; *p* = 0.013; *I* ^2^ = 0.0%), and TC (WMD: −9.70 mg/dL; 95% CI: −14.95, −4.46; *p* < 0.001; *I* ^2^ = 38.3%) in adults. However, okra showed no notable impact on HOMA‐IR, HDL‐C, and LDL‐C. Okra consumption has an improving effect on adult FBG, HbA1c, TG, and TC levels. More large‐scale RCT studies are necessary to validate the beneficial effects of okra on adults due to the limited number of included RCTs.

**Trial Registration:** PROSPERO: CRD42024540121

## Graphical abstract

Okra supplementation significantly reduced adults' FBG, HbA1c, TG, and TC. Okra supplementation had no significant effect on HOMA‐IR, HDL‐C, and LDL‐C. Okra supplementation has an ameliorating effect on FBG, HbA1c, TG, and TC levels in adults.

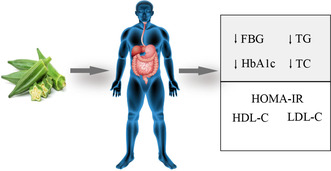

**Abbreviations**
CI: confidence interval
FBG: fasting blood glucose
HbA1c: glycosylated hemoglobin
HDL‐C: high‐density lipoprotein cholesterol
HOMA‐IR: homeostatic model assessment for insulin resistance
LDL‐C: low‐density lipoprotein cholesterol
RCTs: randomized controlled trials
SD: standard deviation
SE: standard error
TC: total cholesterol
TG: triglyceride
WMD: weighted mean difference

## 1 Introduction

Cardiovascular disease (CVD) stands as the primary global cause of human mortality (Zhang et al. 2022). In 2019, cardiovascular‐related diseases claimed the lives of over 17.9 million individuals worldwide, with projections indicating a surge to over 23.6 million deaths by 2030 (Huang et al. 2023). Key contributors to CVD comprise overweight, obesity, diabetes, hypertension, insulin resistance, and dyslipidemia (Kelishadi et al. 2022). Blood glucose and lipids represent modifiable risk elements for CVD (Hong et al. 2017). Therefore, controlling these risk factors that may lead to CVD can effectively prevent or reduce its incidence (Hong et al. 2017). Studies have shown that intervening in CVD‐related risk factors through dietary or nutritional therapy is an effective strategy for forestalling CVD occurrence (Huang et al. 2020; Rahnama et al. 2023; Tierney et al. 2020).

Okra ( *Abelmoschus esculentus* L.) is a vegetable widely distributed in Southern Europe, America, Africa, and Asia (Chowdhury et al. 2019). In traditional medicine, okra is commonly employed to alleviate spasms, treat diabetes, promote diuresis, and cool the body (Islam 2019; Roy, Shrivastava, and Mandal 2014). Rich in bioactive fibers, polysaccharides, and antioxidant components, okra qualifies as a functional food (Agregán et al. 2023). Various studies demonstrate the antioxidant, anti‐diabetes, anti‐hyperlipidemia, immune regulation, anti‐inflammatory, and antibacterial pharmacological activities of okra (Abdel‐Razek et al. 2023). Preclinical studies showcase okra's ability to improve blood glucose and lipid irregularities (Fan et al. 2014; Kzar et al. 2019; Mondal, Gowda, and Manandhar 2019; Nguekouo et al. 2018; Peter et al. 2021). Some clinical studies have also examined the impact of okra on blood glucose and lipid levels in adults with diverse health statuses, and have reached inconsistent conclusions regarding the effects of okra on FBG, HbA1c, TG, TC, and HDL‐C (Afsharmanesh et al. 2024; Bahreini et al. 2024; Nikpayam Saghafi‐Asl et al. 2024; Tavakolizadeh et al. 2023). A systematic review conducted by (Nikpayam Safaei, Bahreini, and Saghafi‐Asl 2021) included two clinical studies and 52 animal studies, indicating an improving effect of okra on hyperlipidemia and hyperglycemia. In addition, a meta‐analysis conducted by (Mokgalaboni et al. 2023) encompassing eight clinical studies, investigated the influence of okra on FBG and HbA1c in patients with type 2 diabetes mellitus (T2DM) and prediabetes. Mokgalaboni's study included randomized and non‐randomized clinical trials and a study with an intervention duration of no more than 1 week, which found that okra only improved FBG and had no effect on HbA1c (Mokgalaboni et al. 2023).

Given the inconsistent findings of previous studies on the effects of okra on blood glucose and lipid levels, we conducted this study to explore the impact of okra on glycemic parameters and lipid profiles in adults through a meta‐analysis of relevant RCT studies.

## 2 Methods

This study was conducted based on the statement of Preferred Reporting Items of Systematic Reviews and Meta‐Analysis (PRISMA) 2020 (Page et al. 2021).

### 2.1 Search Strategy

Two reviewers (X.L. Z. and J.X. M.) systematically searched relevant RCTs in databases Embase, Web of Science, Cochrane Library, Scopus, and PubMed based on established search terms, from inception to April 2024. The search terms include “ *Abelmoschus esculentus* ”, “ *Hibiscus esculentus* ”, “Abelmoschus”, “esculentus”, and “Okra”, in combination with “randomized controlled trial”, “randomized controlled trials”, “clinical trial”, “clinical trials”, “random”, “random allocation”, “placebo”, “placebos”, and “controlled clinical trial” (Table S1). No language restriction was applied during the retrieval process. The references of included studies and relevant articles were also reviewed to avoid missing any studies.

### 2.2 Study Selection

Two reviewers (X.L. Z. and J.X. M.) independently screened the retrieved studies based on inclusion and exclusion criteria, with a third reviewer (Y.G. S.) responsible for resolving their disagreements. Cohen's kappa coefficient was used to evaluate the consistency between the two reviewers (Cohen's kappa = 0.894). The inclusion criteria encompass (a) RCTs performed on adults (age > 18 years); (b) okra as the intervention measure and placebo as the control; (c) reported at least one of the following outcomes data pre‐ and post‐intervention: HOMA‐IR, HbA1c, FBG, TG, TC, LDL‐C, and HDL‐C. The exclusion criteria included: (a) intervention duration < 1 week; (b) insufficient outcome data information; (c) combined application with other herbal components; (d) animal studies, conference abstracts, reviews, case reports, commentaries, or letters to editors.

### 2.3 Data Extraction

A structured data collection table was utilized to extract data from eligible studies. The data of eligible studies were extracted independently by two reviewers (X.L. Z. and J.X. M.), with a third reviewer (Y.G. S.) responsible for resolving any disagreements (Cohen's kappa = 0.918). The extracted data included basic information on articles and patients, pre‐ and post‐intervention levels of HOMA‐IR, HbA1c, FBG, TG, TC, LDL‐C, and HDL‐C.

### 2.4 Quality Assessment

Two reviewers (X.L. Z. and J.X. M.) independently utilized the Cochrane Collaboration risk of bias tool to evaluate the included studies' bias risk (Higgins et al. 2011). The assessment of the risk of bias was mainly based on the following aspects: (a) random sequence generation; (b) allocation concealment; (c) blinding of participants and personnel; (d) blinding of outcome assessment; (e) incomplete outcome data; (f) selective reporting; (g) other bias. The quality of evidence was assessed using the GRADE (Grading of Recommendations Assessment, Development, and Evaluation) approach based on five aspects: risk of bias, inconsistency, indirectness, imprecision, and publication bias, with four levels of certainty: high, moderate, low, and very low (Guyatt et al. 2011). In cases of disagreement, the third reviewer (Y.G. S.) resolved the conflicts between the reviewers (X.L. Z. and J.X. M.) (Cohen's kappa = 0.905).

### 2.5 Statistical Analysis

This study used Stata 17.0 software to conduct the meta‐analysis. The mean change and standard deviation (SD) pre‐ and post‐intervention were employed for quantity analysis of each outcome indicator. The following equation was applied to calculate the SD of the mean change: ${\mathrm{SD}}^{2}\text{change}=S{D^{2}}_{\mathrm{pre}}+S{D^{2}}_{\text{post}}-2R\;\times \;SD_{\mathrm{pre}}\times \;SD_{\text{post}}$, *R* = 0.5 (Borenstein et al. 2009). A formula $\mathrm{SD}=\mathrm{SE}\times \sqrt{N}$ was used to calculate SD when a standard error (SE) was reported (Hozo, Djulbegovic, and Hozo 2005). The pooled effect size was presented with a weighted mean difference (WMD) and its 95% confidence interval (CI). The *p* < 0.05 was considered to have statistical differences. The Higgins *I* ^2^ statistic was used to assess the heterogeneity among studies. If *I* ^2^ ≥ 50% and *p*‐value < 0.05, the heterogeneity was considered high and a random‐effects model was chosen (Higgins et al. 2003). Otherwise, a fixed‐effects model was applied. The TG, TC, LDL‐C, HDL‐C, and FBG units were converted from mmol/L to mg/dL. Subgroup analyses were carried out categorizing by intervention type, duration, dose, and health status to pinpoint potential sources of heterogeneity. Egger's and Begg's tests were applied to evaluate publication bias. Sensitivity analysis was conducted to assess the impact of each study on the merged results. Due to the limited number of studies included (< 10), a funnel plot was not employed to detect publication bias (Egger et al. 1997).

## 3 Results

### 3.1 Study Selection

**FIGURE 1:**
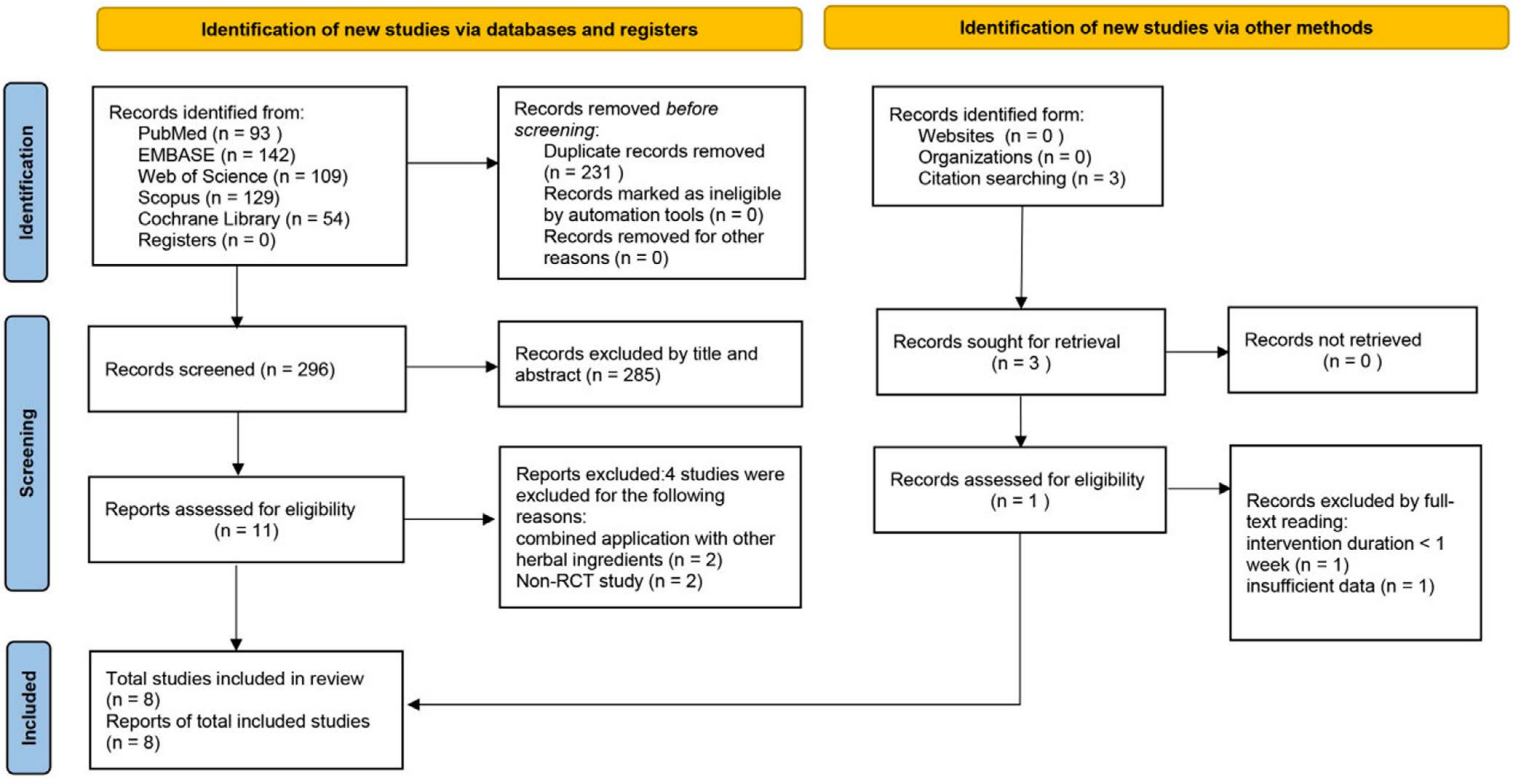
Flowchart of included study selection.

A total of 527 studies were identified during the search process. Of these, 231 were removed for duplicates. Following the inclusion and exclusion criteria, 285 studies were excluded by reading the title and abstract, and the remaining 11 were for full‐text review. Out of the remaining 11 studies, four were excluded for the following reasons: combining application with other herbal ingredients (*n* = 2); and non‐RCT research (*n* = 2). Three articles were retrieved through reference review, while two were excluded after full‐text reading because of intervention duration < 1 week (*n* = 1) or reporting insufficient data (*n* = 1). Ultimately, 8 studies (with 9 arms) were included in the meta‐analysis (Figure 1).

### 3.2 Studies Characteristics

Table 1 outlines the basic information of the included studies. The eight included studies were published from 2020 to 2024 (Afsharmanesh et al. 2024; Bahreini et al. 2024; Khodija, Wiboworini, and Kartikasari 2020; Moradi et al. 2020; Nikpayam Saghafi‐Asl et al. 2024; Saatchi et al. 2022; Salarfard et al. 2023; Tavakolizadeh et al. 2023). One study was a non‐blinded trial (Salarfard et al. 2023), and one trial did not report blinding (Khodija, Wiboworini, and Kartikasari 2020). Among the remaining six studies, two were triple‐blinded trials (Bahreini et al. 2024; Nikpayam Saghafi‐Asl et al. 2024), and four were double‐blinded trials (Afsharmanesh et al. 2024; Moradi et al. 2020; Salarfard et al. 2023; Tavakolizadeh et al. 2023). Seven trials were conducted in Iran (Afsharmanesh et al. 2024; Bahreini et al. 2024; Moradi et al. 2020; Nikpayam Saghafi‐Asl et al. 2024; Saatchi et al. 2022; Salarfard et al. 2023; Tavakolizadeh et al. 2023), and one in Indonesia (Khodija, Wiboworini, and Kartikasari 2020). The eight studies summed up 521 participants, with a minimum sample size of 40 (Khodija, Wiboworini, and Kartikasari 2020) and a maximum sample size of 99 (Saatchi et al. 2022). Except for one study with only female participants (Salarfard et al. 2023), the remaining seven studies were participants of both genders (Afsharmanesh et al. 2024; Bahreini et al. 2024; Khodija, Wiboworini, and Kartikasari 2020; Moradi et al. 2020; Nikpayam Saghafi‐Asl et al. 2024; Saatchi et al. 2022; Tavakolizadeh et al. 2023). The health status of participants involves T2DM, prediabetes, diabetic nephropathy, and gestational diabetes mellitus, with an average age of 28.0–64.6 years. Khodija's study with two intervention arms was defined as respectively (Khodija, Wiboworini, and Kartikasari 2020). Two studies intervened with 80 mg of okra extract daily (Bahreini et al. 2024; Nikpayam Saghafi‐Asl et al. 2024), while six studies intervened with 3–40 g of okra daily (Afsharmanesh et al. 2024; Khodija, Wiboworini, and Kartikasari 2020; Moradi et al. 2020; Saatchi et al. 2022; Salarfard et al. 2023; Tavakolizadeh et al. 2023). The intervention duration ranges from 2 to 12 weeks.

**TABLE 1:** Characteristics of the included studies.

Author	Year	Country	Study design	Participants gender	Health status	Mean age (years)	Sample size for analysis	Intervention type	Daily dose	Intervention duration	Control group	Outcomes
						I/C	I/C					
Afsharmanesh et al.	2024	Iran	Double‐blinded	M/F	Prediabetes	45.81 ± 6.59, 45.61 ± 7.80	35/35	Okra	3 g	8 weeks	Placebo	TG, TC, HDL‐C, HDL‐C
Bahreini et al.	2024	Iran	Triple‐blinded	M/F	Diabetic nephropathy	62 ± 7, 64.6 ± 8.5	30/25	Okra extract	80 mg	10 weeks	Placebo	TG, TC, HDL‐C, HDL‐C
Khodijaet al.	2020a	Indonesia	NR	M/F	T2DM with hypercholesterolemia	NR	12/8	Boiled okra	40 g	2 weeks	Placebo	FBG
Khodijaet al.	2020b	Indonesia	NR	M/F	T2DM with hypercholesterolemia	NR	12/8	Steamed okra	40 g	2 weeks	placebo	FBG
Moradi et al.	2020	Iran	Double‐blinded	M/F	T2DM	54.26 ± 7.62, 53.33 ± 7.35	25/23	Okra	10 g	8 weeks	Placebo	FBG, HbA1c, HOMA‐IR, TG, TC, HDL‐C, HDL‐C
Nikpayam et al.	2024	Iran	Triple‐blinded	M/F	Diabetic nephropathy	62 ± 7, 61.6 ± 8.5	30/25	Okra extract	80 mg	10 weeks	Placebo	FBG, HbA1c, HOMA‐IR
Saatchi et al.	2022	Iran	Double‐blinded	M/F	T2DM	57.7 ± 9.7, 58.3 ± 9.2	50/49	Okra	4 g	8 weeks	Placebo	FBG, HbA1c, TG, TC
Salarfard et al.	2023	Iran	Non‐blinded	F	Gestational diabetes mellitus	29.0 ± 3.9, 28.0 ± 4.6	30/30	Okra	6 g	4 weeks	Placebo	FBG
Tavakolizadeh et al.	2023	Iran	Double‐blinded	M/F	T2DM	53.8 ± 3.7, 52.8 ± 4.6	48/46	Okra	3 g	12 weeks	Placebo	FBG, HbA1c, HOMA‐IR, TG, TC

Abbreviations: C, control; F, female; I, intervention; M, male; NR, not reported; T2DM, type 2 diabetes mellitus.

### 3.3 Risk of Bias Assessment

**FIGURE 2:**
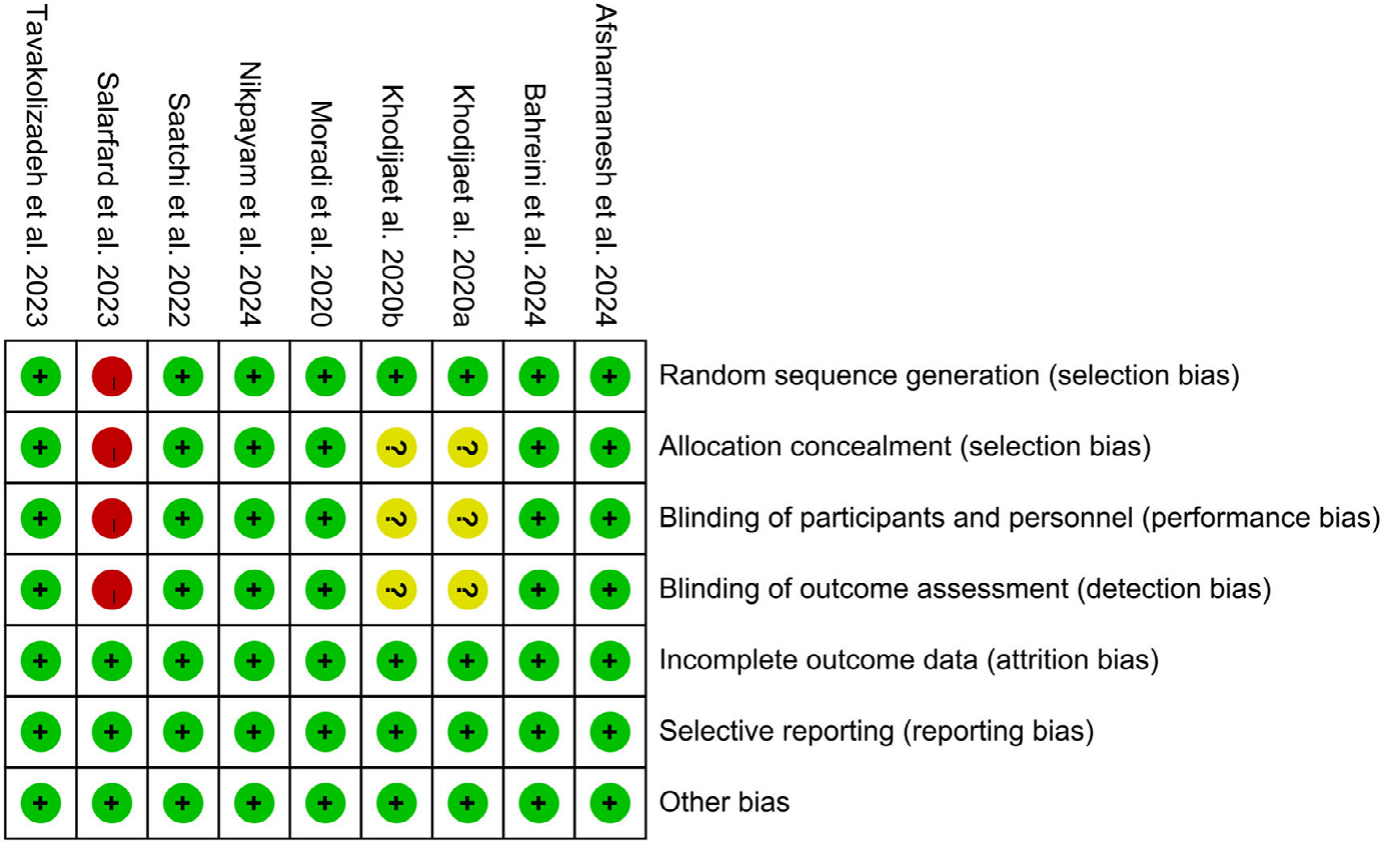
Risk of bias assessment of included studies. A green dot accompanied by a plus sign signifies a low risk of bias, whereas a yellow dot with a question mark indicates an unclear risk of bias, and a red dot with a minus sign denotes a high risk of bias.

Due to the absence of blinding during the research process, one study was assessed as high risk in random sequence generation, allocation concealment, blinding of participants and personnel, and blinding of outcome assessment (Salarfard et al. 2023). Kohodija's study was evaluated as unclear regarding allocation concealment, blinding of participants and personnel, and blinding of outcome assessment due to a lack of relevant information (Khodija, Wiboworini, and Kartikasari 2020). In addition, all other studies were evaluated as having low‐risk bias. Eventually, the overall risk of bias was assessed as low risk (Figure 2).

### 3.4 Findings From the Systematic Review

Six studies (with seven arms) evaluated FBG levels (Khodija, Wiboworini, and Kartikasari 2020; Moradi et al. 2020; Nikpayam Saghafi‐Asl et al. 2024; Saatchi et al. 2022; Salarfard et al. 2023; Tavakolizadeh et al. 2023), of which five studies (with six arms) demonstrated notably decreased FBG levels after okra intervention (Khodija, Wiboworini, and Kartikasari 2020; Nikpayam Saghafi‐Asl et al. 2024; Saatchi et al. 2022; Salarfard et al. 2023; Tavakolizadeh et al. 2023). Among the four studies evaluating HbA1c (Moradi et al. 2020; Nikpayam Saghafi‐Asl et al. 2024; Saatchi et al. 2022; Tavakolizadeh et al. 2023), two showed a remarkable decrease in HbA1c after okra treatment (Saatchi et al. 2022; Tavakolizadeh et al. 2023). Out of three studies evaluating HOMA‐IR (Moradi et al. 2020; Nikpayam Saghafi‐Asl et al. 2024; Tavakolizadeh et al. 2023), none observed a significant impact of okra consumption on HOMA‐IR. Five studies assessed okra's effect on TG and TC levels (Afsharmanesh et al. 2024; Bahreini et al. 2024; Moradi et al. 2020; Saatchi et al. 2022; Tavakolizadeh et al. 2023). Three studies indicated that okra significantly reduced TC levels (Afsharmanesh et al. 2024; Moradi et al. 2020; Tavakolizadeh et al. 2023), while one showed that okra decreased TG levels (Moradi et al. 2020). Three studies assessed okra's effect on HDL‐C and LDL‐C levels (Afsharmanesh et al. 2024; Bahreini et al. 2024; Moradi et al. 2020). No studies indicated that okra dramatically reduced LDL‐C levels, while only one study revealed that okra increased HDL‐C levels (Afsharmanesh et al. 2024).

### 3.5 Effects of Okra Consumption on Glycemic Parameters

**FIGURE 3:**
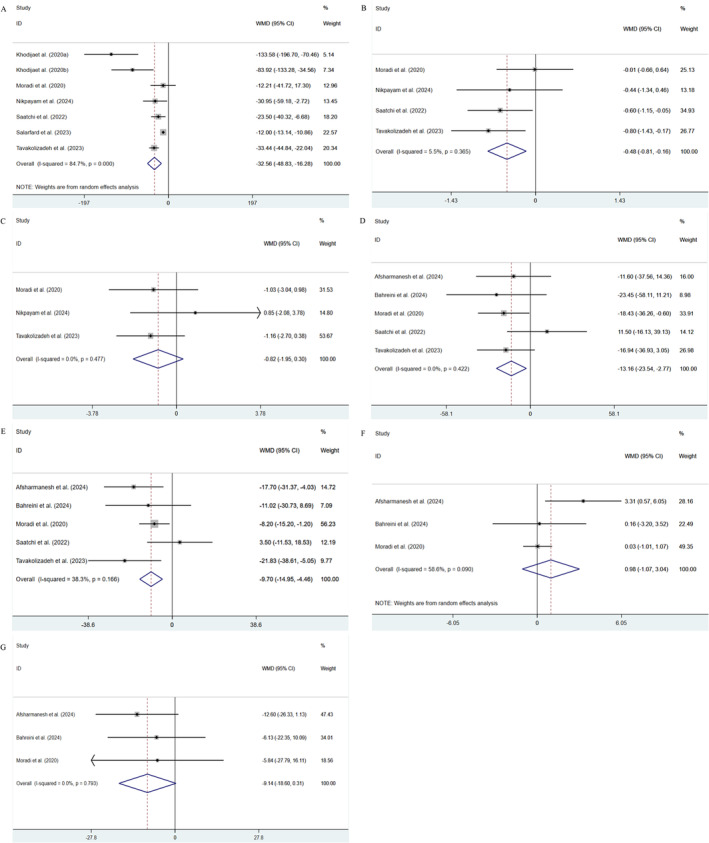
Forest plot of the effects of okra consumption of (A) FBG (mg/dL), (B) HbA1c (%), (C) HOMA‐IR, (D) TG (mg/dL), (E) TC (mg/dL), (F) HDL‐C (mg/dL), and (G) LDL‐C (mg/dL).

Six studies (with seven arms, and 396 participants) evaluated the impact of okra on FBG. The results revealed that, following the okra intervention, there was a remarkable decrease in FBG levels compared to the placebo (WMD: −32.56 mg/dL; 95% CI: −48.83, −16.28; *p <* 0.001), with high heterogeneity (*I* ^2^ = 84.7%, *p* < 0.001) (Figure 3A). No potential source of heterogeneity was identified through subgroup analysis (Table 2). Sensitivity analysis indicated the robustness of the pooled results by omitting studies one by one (Figure S1).

**TABLE 2:** Subgroup analysis of the effects of okra consumption on FBG.

	Meta‐analysis			Heterogeneity		
Study group	Number of studies	WMD (95% Cl)	*p*‐effect	*I* ^2^	*p*‐within group	*p*‐between group
Intervention type						
Okra	6	−33.23 (−51.22, −15.25)	< 0.001	86.6%	< 0.001	0.673
Okra extract	1	−30.95 (−59.18, −2.72)	0.032	—	—	
Intervention duration						
< 8 weeks	3	−71.71 (−147.88, 4.45)	0.065	91.1%	< 0.001	0.343
≥ 8 weeks	4	−28.84 (−37.41, −20.28)	< 0.001	0.0%	0.521	
Intervention dose						
< 3 g/day	1	−30.95 (−59.18, −16.28)	0.032	—	—	0.972
≥ 3 g/day	6	−33.23 (−51.22, −15.25)	< 0.001	86.6%	< 0.001	
Health status						
T2DM	5	−36.40 (−60.33, −12.47)	0.003	83.4%	< 0.001	0.804
Non‐T2DM	2	−33.09 (−43.66, −22.52)	< 0.001	0.0%	0.873	

Four studies (involving 296 participants) evaluated the effect of okra consumption on HbA1c. The pooled results of a fixed‐effects model indicated a significant reduction of HbA1c levels after okra consumption (WMD: −0.48%; 95% CI: −0.81, −0.16; *p* = 0.004), with no obvious heterogeneity (*I* ^2^ = 5.5%, *p* = 0.365) (Figure 3B). Given the limited number of studies, subgroup analysis was not performed. Sensitivity analysis indicated that removing any single study did not significantly change the overall outcomes (Figure S1).

Three trials (with 197 participants) examined the impact of okra consumption on HOMA‐IR. The results revealed that no notable decrease was observed in HOMA‐IR following the intervention of okra compared with the placebo (WMD: −0.08; 95% CI: −1.95, 0.30; *p* = 0.153) (Figure 3C). Additionally, no heterogeneity was observed among the studies (*I* ^2^ = 0.0%, *p* = 0.477). Subgroup analysis was not conducted due to the limited number of studies. The robustness of the pooled outcomes was demonstrated by sensitivity analysis (Figure S1).

### 3.6 Effects of Okra Consumption on Lipid Profile

Four studies (with 366 participants) assessed the effects of okra consumption on TG and TC. The combined results indicated a remarkable decrease in TG (WMD: −13.16 mg/dL; 95% CI: −23.54, −2.77; *p* = 0.013) and TC (WMD: −9.70 mg/dL; 95% CI: −14.95, −4.46; *p* < 0.001) following okra intervention (Figure 3D,E). No significant heterogeneity was noted in TG (*I* ^2^ = 0.0%, *p* = 0.422) and TC (*I* ^2^ = 38.3%, *p* = 0.166). Three studies (with 173 participants) evaluated the impact of okra on HDL‐C and LDL‐C. The findings showed no remarkable difference in HDL‐C (WMD: 0.98 mg/dL; 95% CI: −1.07, 3.04; *p* = 0.349) and LDL‐C (WMD: −9.14 mg/dL; 95% CI: −18.60, 0.31; *p* = 0.058), with heterogeneity in HDL‐C (*I* ^2^ = 58.6%; *p* = 0.090) and LDL‐C (*I* ^2^ = 0.0%; *p* = 0.793, Figure 3F,G). Sensitivity analysis demonstrated the robustness of the pooled outcomes in TG, TC, HDL‐C, and LDL‐C (Figure S1). The removal of Afsharmanesh's study resulted in the disappearance of heterogeneity of HDL‐C (*I* ^2^ = 0.0%; *p* = 0.942) and LDL‐C (*I* ^2^ = 0.0%; *p* = 0.999). Subgroup analysis was not conducted for TG, TC, HDL‐C, and LDL‐C due to the limited number of studies.

### 3.7 Quality of Evidence Assessment

Based on indirectness, inconsistency, imprecision, risk of bias, and publication bias, the evidence quality for FBG was rated as “very low” due to imprecision, inconsistency, and risk of bias. Regarding HbA1c, HOMA‐IR, TG, and TC, the quality of evidence was evaluated as “moderate” due to imprecision. A “low” quality was assessed for HDL‐C and LDL‐C due to imprecision and inconsistency (Table S2).

### 3.8 Publication Bias

According to Egger's and Begg's tests, the *p* values of HOMA‐IR, HbA1c, FBG, TG, TC, HDL‐C, and LDL‐C were 0.234, 0.296; 0.776, 0.734; 0.113, 0.089; 0.992, 0.734; 0.675, 1.000; 0.558; 1.000; and 0.441, 1.000; respectively. The results of Egger's and Begg's tests indicated no publication bias.

## 4 Discussion

Our findings indicated that okra consumption significantly reduced FBG, HbA1c, TG, and TC levels in adults based on the included RCT studies. Nevertheless, okra consumption did not dramatically alter HOMA‐IR, HDL‐C, and LDL‐C levels. Afsharmanesh's study might be the source of heterogeneity in HDL‐C. Despite conducting subgroup analysis, we did not identify the sources of FBG heterogeneity. The heterogeneity of FBG may be related to differences in research design and data statistical methods among included studies.

Preclinical investigations have shown that okra and its extracts have anti‐hyperglycemic effects. Okra powder, okra extract, and okra polysaccharide can significantly reduce the elevated FBG level in streptozotocin (STZ) induced diabetes model animals, improving the HOMA‐IR index (Erfani Majd et al. 2018; Husen et al. 2020; Liao et al. 2019; Zuraidah et al. 2019). The extract of okra rich in polysaccharides and carbohydrates can significantly reduce the HbA1c level of diabetes rats induced by a high‐fat diet and STZ (Huang et al. 2017). Consistent with preclinical research results, our findings also demonstrated that okra significantly reduced the levels of FGP and HbA1c in adults. Interestingly, a previous meta‐analysis found that okra could reduce FBG and HbA1c levels in T2DM populations, although some non‐RCT studies were included (Mokgalaboni et al. 2023). Unlike this, we only included RCT studies with intervention duration exceeding 1 week. Our results also indicated that okra had a reducing effect on FBS and HbA1c. In addition, subgroup analysis indicated that both T2DM and non‐T2DM populations could benefit from okra consumption in reducing FPG. However, we found that okra did not have a notable improvement effect on HOMA‐IR. Okra exhibits anti‐hyperglycemic effects through diverse mechanisms including the promotion of damaged insulin cell regeneration, increased insulin secretion, stimulation of liver glycogen synthesis, and reduced glucose absorption in the intestines, effectively lowering blood sugar levels (Abbas et al. 2017). Peroxisome proliferator‐activated receptor (PPAR) is important for maintaining glucose homeostasis and regulating the expression of glucose metabolism‐related genes. Okra can improve β‐Cell impairment and glucose homeostasis by regulating the PPAR‐dependent pathway (Erfani Majd et al. 2018). Furthermore, certain phenolic compounds contained within okra possess inhibitory effects on the activities of α‐amylase, pancreatic lipase, and α‐glucosidase (Shen et al. 2019). Kaempferol contained in okra can improve insulin‐dependent glucose intake in adipocytes and reduce insulin levels (Bhattacharya et al. 2013; Sharma, Kumar Tekade, and Kalia 2020). Okra polysaccharides have been demonstrated to induce GSK3β phosphorylation, maintain glycogen synthase activity, and promote glycogen synthesis by regulating the insulin/PI3K/Akt pathway (Geng et al. 2022). Okra pectin polysaccharides can exert anti‐hyperglycemic effects by inhibiting lipid peroxidation chain reactions (Zhang et al. 2018). Flavonoids such as isoquercitrin and rutin in okra can inhibit the formation of AGEs and non‐enzymatic protein glycation (Zhang and Yan 2023). The quercitrin contained in okra can activate AMPK‐α, promoting the expression of the glucose transporter type 4 (GLUT4) gene and improving insulin resistance (Nasrollahi et al. 2022).

Several preclinical studies have shown that okra powder, okra extract, and okra polysaccharide can also significantly reduce TG, TC, and LDL‐C levels, and increase LDL‐C in diabetic rats (Fan et al. 2014; Husen et al. 2020; Kzar et al. 2019; Liao et al. 2019; Mondal, Gowda, and Manandhar 2019; Nabila, Damayanthi, and Marliyati 2018; Nguekouo et al. 2018; Uadia et al. 2020). However, some clinical studies did not observe any improvement effect of okra on TG, TC, HDL‐C, and LDL‐C levels (Bahreini et al. 2024; Saatchi et al. 2022). Our findings indicated a noteworthy decrease in TG and TC levels following okra consumption, while no significant impact was observed in HDL‐C and LDL‐C. The mechanism of okra's anti‐hyperlipidemic effect includes inhibiting PPARs, inhibiting fat generation by reducing the expression of fatty acid synthase (FAS) and sterol regulatory element‐binding protein 1c (SREBP1c), promoting cholesterol degradation by increasing the expression of cholesterol 7α‐hydroxylase (CYP7A1), inhibiting cholesterol absorption, and inhibiting the binding of cholesterol and bile acids (Esmaeilzadeh, Razavi, and Hosseinzadeh 2020; Wang et al. 2014). However, okra has no impact on low‐density lipoprotein receptor (LDLR), 3‐hydroxy‐3‐methylglutaryl‐CoA reductase (HMGR), carnitine palmitoyltransferase‐1A (CPT1A), and sterol regulatory element binding protein 2 (SREBP2) (Wang et al. 2014).

The current meta‐analysis possesses several strengths. To the best of our knowledge, this study presents the first meta‐analysis of RCTs examining the clinical benefits of okra in adults. This meta‐analysis was conducted based on PRISMA and underwent GRADE evaluation to ensure the quality of evidence. A few limitations of this study need to be considered. Firstly, due to the small number and sample size of included studies, caution should be exercised in interpreting the results of this study. Secondly, we did not conduct a gray literature search, which may have resulted in certain studies being overlooked. However, we reviewed the references of included studies to minimize the omission of relevant studies. Finally, potential sources of high heterogeneity in fasting blood glucose (FBG) were not identified through subgroup analysis, potentially affecting result credibility. Nevertheless, sensitivity analysis confirmed the robustness of the FBG results.

## 5 Conclusion

The current meta‐analysis indicates that okra consumption can improve FBG, HbA1c, TG, and TC levels in adults. However, okra consumption does not dramatically affect the HOMA‐IR, HDL‐C, and LDL‐C levels. More large‐scale RCT studies are needed to confirm the beneficial effects of okra on adults.

## Author Contributions

**Xiaolei Zhang:** conceptualization, methodology, software, data curation, writing – original draft, writing – review and editing. **Jinxin Miao:** data curation, software, writing – original draft, writing – review and editing. **Yagang Song:** data curation, methodology, writing – review and editing, supervision. **Mingsan Miao:** conceptualization, funding acquisition, project administration, writing – review and editing, supervision.

## Conflicts of Interest

The authors declare no conflicts of interest.

## Supporting information


Figure S1.

Table S1.

Table S2.

## Data Availability

The data that support the findings of this study are available on request from the corresponding author.
