# Supplementary material for: The Effects of Okra Consumption on Glycemic Parameters and Lipid Profile in Adults: A Systematic Review and Meta‐Analysis
Source: Food Sci Nutr. 2024 Nov 20;12(12):10049–58. doi: 10.1002/fsn3.4599 (PMC11666828; doi:10.1002/fsn3.4599)
Supplement: Supplementary file 1 — Figure S1. [file FSN3-12-10049-s002.docx]

| A | 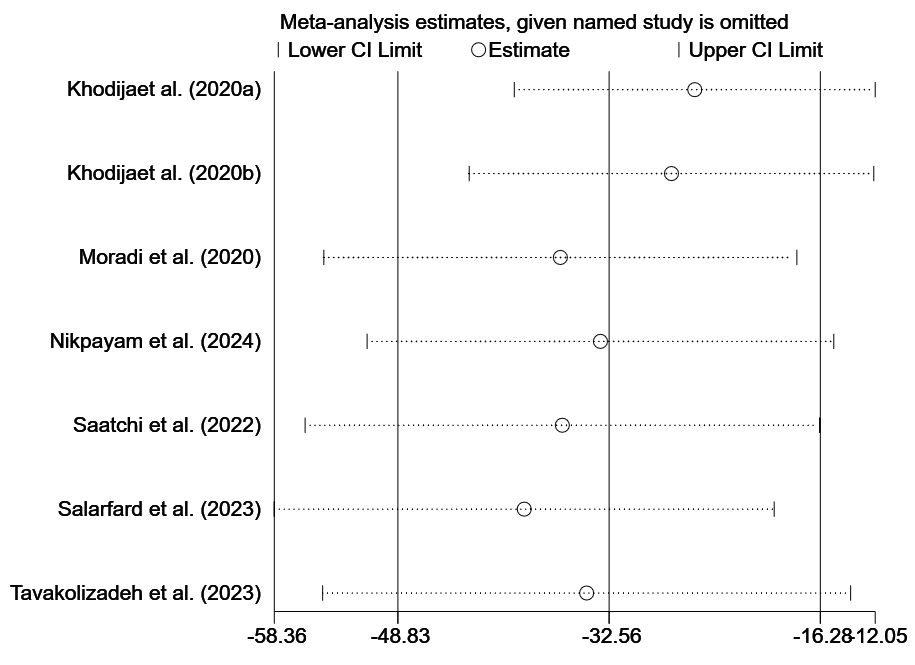 |
| --- | --- |
| B | 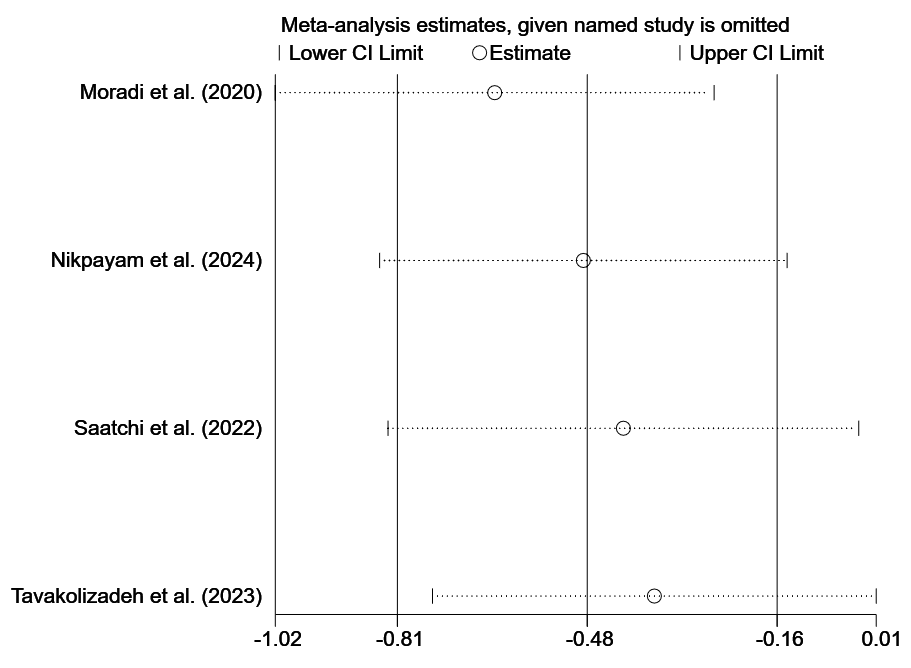 |
| C | 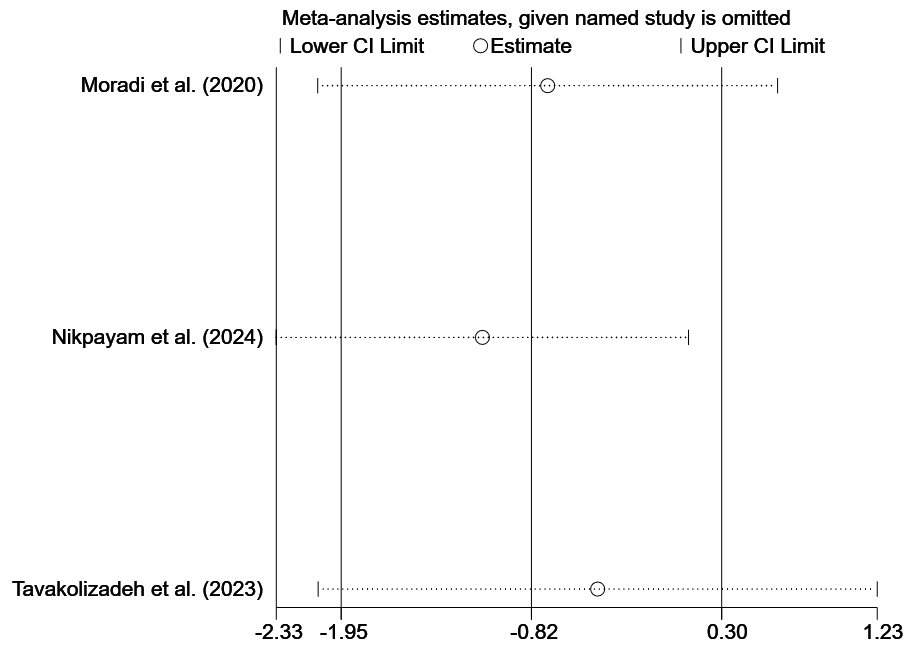 |
| D | 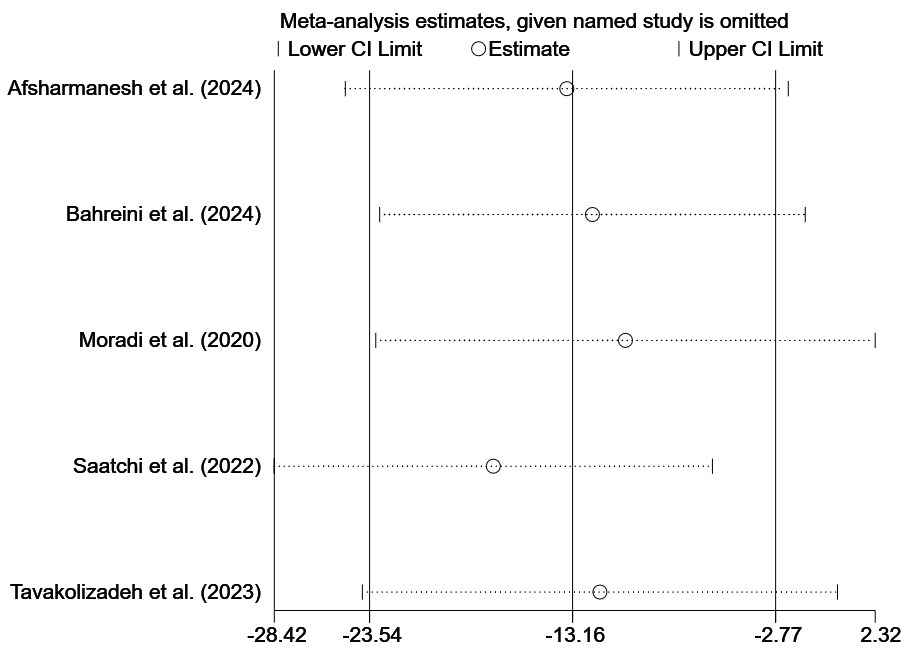 |
| E | 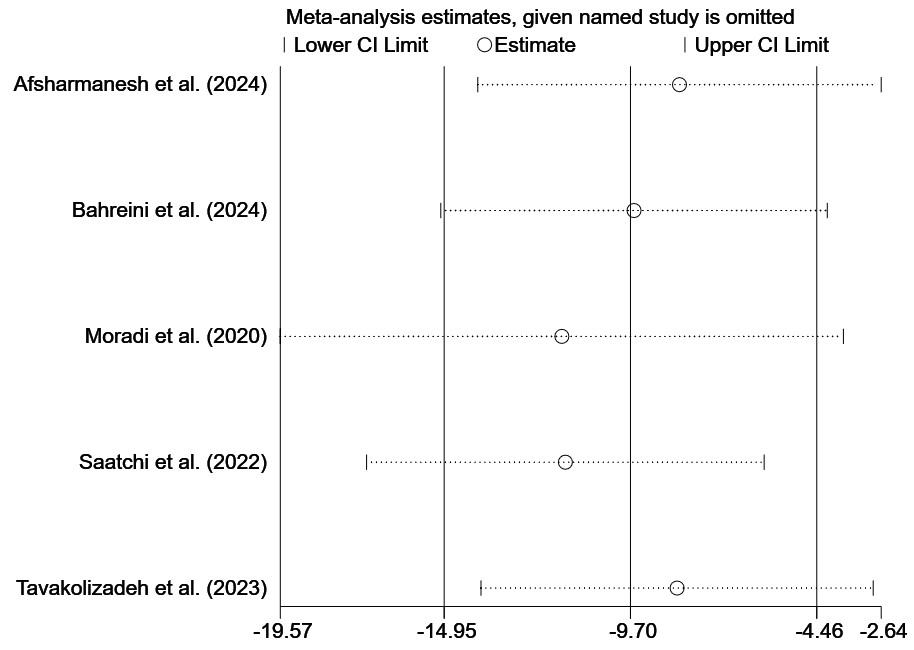 |
| F | 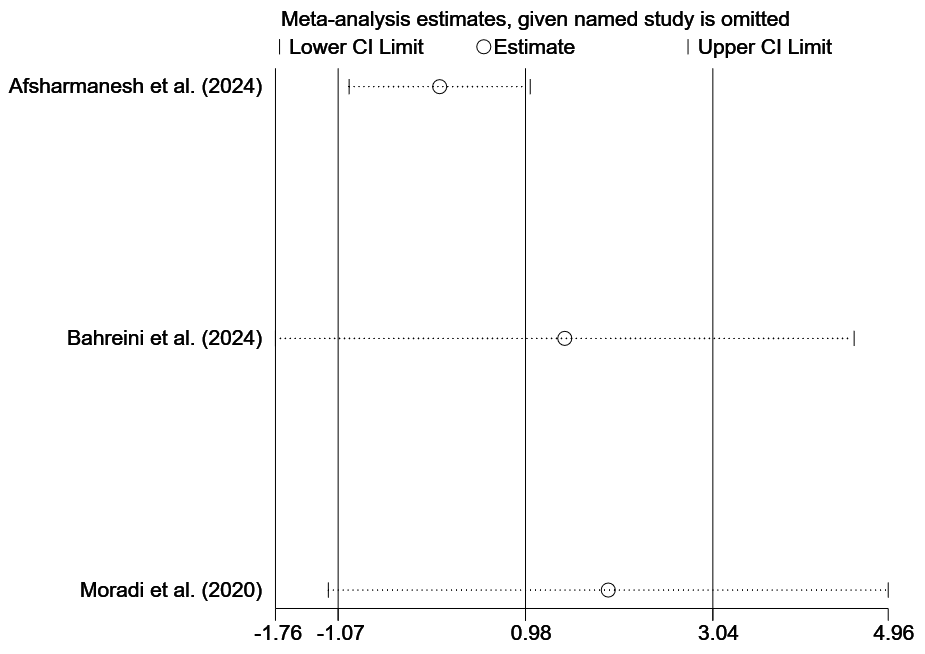 |
| G | 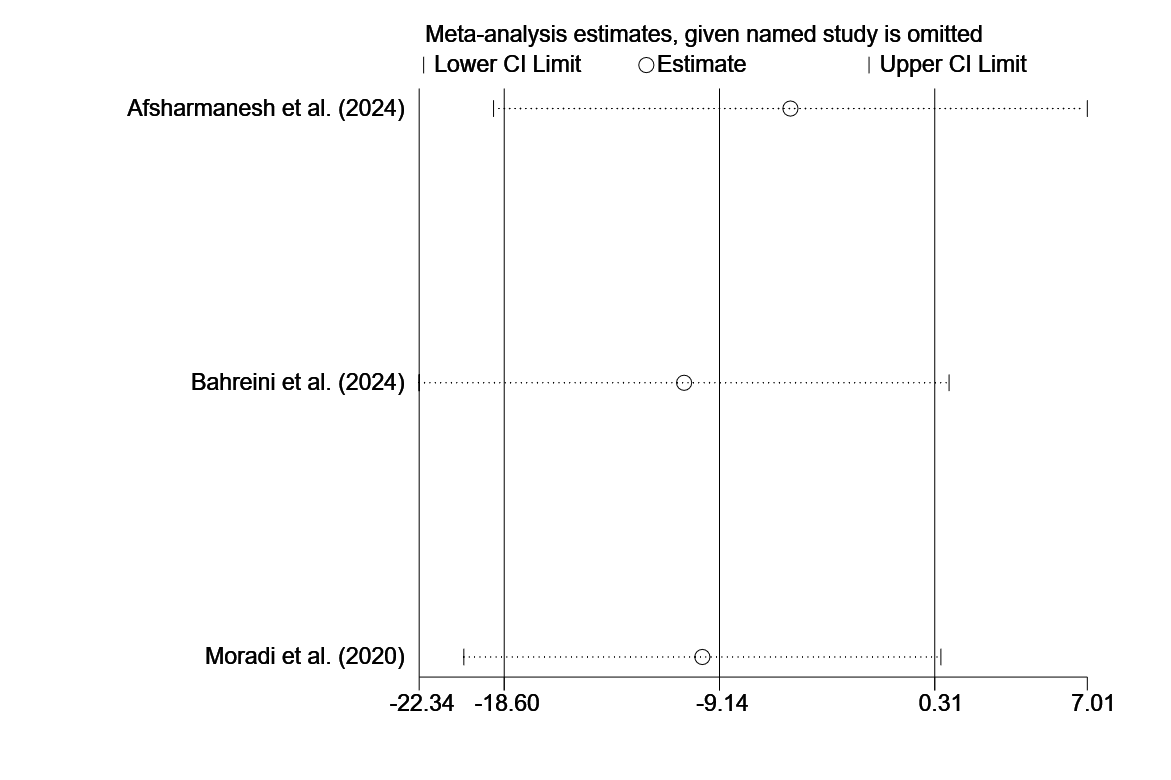 |
| **Supplementary Fig.1.** Sensitivity analysis of the effects of okra consumption on FBG (A), HbA1c (B), HOMA-IR (C), TG (D), TC (E), HDL-C (F), and LDL-C (G). | |
