# Supplementary material for: The Effects of Okra Consumption on Glycemic Parameters and Lipid Profile in Adults: A Systematic Review and Meta‐Analysis
Source: Food Sci Nutr. 2024 Nov 20;12(12):10049–58. doi: 10.1002/fsn3.4599 (PMC11666828; doi:10.1002/fsn3.4599)
Supplement: Supplementary file 2 — Table S1. [file FSN3-12-10049-s003.docx]

**Supplementary Table 1.** Full search strategy for PUBMED,EMBASE, Web of science, Scopus, and Cochrane Library.

| **Database** | **Descriptors** | | Results |
| --- | --- | --- | --- |
| **PUBMED** | **#1** | "Abelmoschus"[MeSH Terms] OR "Okra"[Title/Abstract] OR "abelmoschus esculentus"[Title/Abstract] OR "hibiscus esculentus"[Title/Abstract] OR "esculentus"[Title/Abstract] | 93 |
|  | **#2** | "intervention"[Text Word] OR "controlled trial"[Text Word] OR "randomized clinical trial"[Text Word] OR "randomized controlled trial"[Text Word] OR "trial"[Text Word] OR "clinical trial"[Text Word] OR "randomized"[Text Word] OR "random"[Text Word] OR "randomly"[Text Word] OR "placebo"[Text Word] OR "RCT"[Text Word] |  |
|  | **#3** | **#1** AND **#2** |  |
| **EMBASE** | **#1** | 'Abelmoschus':ab,ti OR 'Okra':ab,ti OR 'abelmoschus esculentus':ab,ti OR 'hibiscus esculentus':ab,ti OR 'esculentus':ab,ti | 142 |
|  | **#2** | 'randomized clinical trial':ab,ti OR 'randomized controlled trial':ab,ti OR 'controlled trial':ab,ti OR 'clinical trial':ab,ti OR 'trial':ab,ti OR 'intervention':ab,ti OR 'randomized':ab,ti OR 'random':ab,ti OR 'randomly':ab,ti OR 'placebo':ab,ti OR 'rct':ab,ti |  |
|  | **#3** | **#1** AND **#2** |  |
| **Web of Science** | **#1** | TS= ("Abelmoschus" OR "Okra" OR "abelmoschus esculentus" OR "hibiscus esculentus" OR "esculentus") | 109 |
|  | **#2** | TS=("randomized controlled trial" OR "controlled clinical trial" OR "randomized controlled trials" OR "random allocation" OR "triple blind method" OR "double blind method" OR "single blind method" OR "clinical trial" OR "clinical trials" OR "placebos" OR "placebo" OR "random") |  |
|  | **#3** | **#1** AND **#2** |  |
| **Scopus** | **#1** | TITLE-ABS-KEY ( "Abelmoschus" OR "Okra" OR "abelmoschus esculentus" OR "hibiscus esculentus" OR "h esculentus" ) | 129 |
|  | **#2** | TITLE-ABS-KEY ( "randomized controlled trial" OR "controlled clinical trial" OR "randomized controlled trials" OR "random allocation" OR "triple blind method" OR "double blind method" OR "single blind method" OR "clinical trial" OR "clinical trials" OR "placebos" OR "placebo" OR "random" ) |  |
|  | **#3** | **#1 and #2** |  |
| **Cochrane Library** | **#1** | ("Abelmoschus" OR "Okra" OR "abelmoschus esculentus" OR "hibiscus esculentus" OR "esculentus"):ti,ab,kw | 54 |
