# Supplementary material for: The Effects of Okra Consumption on Glycemic Parameters and Lipid Profile in Adults: A Systematic Review and Meta‐Analysis
Source: Food Sci Nutr. 2024 Nov 20;12(12):10049–58. doi: 10.1002/fsn3.4599 (PMC11666828; doi:10.1002/fsn3.4599)
Supplement: Supplementary file 3 — Table S2. [file FSN3-12-10049-s001.docx]

**Supplementary Table 2.** GRADE profile of okra consumption on glycemic parameters and lipid profile in adults.

| **Certainty assessment** | | | | | | | **Summary of findings** | | | |
| --- | --- | --- | --- | --- | --- | --- | --- | --- | --- | --- |
| **Indicators** | **Number of studies** | **Risk of bias** | **Inconsistency** | **Indirectness** | **Imprecision** | **Other considerations** | **Number of intervention/Control** | **WMD (95% CI)** | **Heterogeneity (*I*^2^ %)** | **Quality of evidence** |
| FPG | 7 | Serious^a^ | Serious^b^ | Not serious | Serious^c^ | None | 207/189 | -32.56 (-48.83, -16.28) | 84.7% | ⨁◯◯◯Very Low |
| HbA1c | 4 | Not serious | Not serious | Not serious | Serious^c^ | None | 153/143 | -0.48 (-0.81, -0.16) | 5.5% | ⨁⨁⨁◯ Moderate |
| HOMA-IR | 3 | Not serious | Not serious | Not serious | Serious^c^ | None | 103/94 | -0.08 (-1.95, 0.30) | 0.0% | ⨁⨁⨁◯ Moderate |
| TG | 5 | Not serious | Not serious | Not serious | Serious^c^ | None | 188/178 | -13.16 (-23.54, -2.77) | 0.0% | ⨁⨁⨁◯ Moderate |
| TC | 5 | Not serious | Not serious | Not serious | Serious^c^ | None | 188/178 | -9.70 (-14.95, -4.46) | 38.3% | ⨁⨁⨁◯ Moderate |
| HDL-C | 3 | Not serious | Serious^b^ | Not serious | Serious^c^ | None | 90/83 | 0.98 (-1.07, 3.04) | 58.6% | ⨁⨁◯◯ Low |
| LDL-C | 3 | Not serious | Serious^b^ | Not serious | Serious^c^ | None | 90/83 | -1.62 (-9.42, 6.19) | 71.9% | ⨁⨁◯◯ Low |

^a^Certain studies have the risk of bias.

^b^High heterogeneity.

^c^Small sample bias may exist.
